# Supplementary material for: Anapole mediated giant photothermal nonlinearity in nanostructured silicon
Source: Nat Commun. 2020 Jun 15;11:3027. doi: 10.1038/s41467-020-16845-x (PMC7296001; doi:10.1038/s41467-020-16845-x)
Supplement: Supplementary file 1 — Supplementary Information [file 41467_2020_16845_MOESM1_ESM.pdf]

Supplementary information

Anapole Mediated Giant Photothermal Nonlinearity in Nanostructured Silicon

Tianyue Zhang[1, #], Ying Che[1, 2, #], Kai Chen[1], Jian Xu[1], Yi Xu[3], Te Wen[4], Guowei Lu[4], Xiaowei Liu[1], Bin Wang[2], Xiaoxuan Xu[2], Yi-Shiou Duh[5], Yu-Lung Tang[5], Jing Han[1], Yaoyu Cao[1], Bai-ou Guan[1], Shi-Wei Chu[5, *] & Xiangping Li[1, *]

[1] Guangdong Provincial Key Laboratory of Optical Fiber Sensing and Communications, Institute of Photonics Technology, Jinan University, Guangzhou 510632, China

[2]The Key Laboratory of Weak-Light Nonlinear Photonics, Ministry of Education, School of Physics, Nankai University, Tianjin 300071, China

[3] Department of Electronic Engineering, College of Information Science and Technology, Jinan University, Guangzhou 510632, China

[4] State Key Laboratory for Mesoscopic Physics, Frontiers Science Center for Nano-optoelectronics & Collaborative Innovation Center of Quantum Matter, School of Physics, Peking University, Beijing 100871, China

[5]Department of Physics, National Taiwan University, No. 1, Sec. 4, Roosevelt Rd., Taipei 10617, Taiwan

[#] These authors contributed equally

*Corresponding author: xiangpingli@jnu.edu.cn; swchu@phys.ntu.edu.tw

Supplementary Note 1: Laser treatment of as-prepared Si nanodisks

The as-prepared nanodisks are amorphous Si which was found to undergo phase transformation when the laser power exceeded 0.5mW (~ 0.3 MW/cm^2). The appearance of a Raman peak at 520 cm^{-1} in the laser annealed sample implies the onset of crystallization. On the other hand, for the a-Si nanodisks without laser annealing, the Raman spectrum contains only a broad band around 475cm^{-1} (Supplementary Figure 1). Therefore, before nonlinear measurement, our Si nanodisks were pre-annealed with relative high laser power of 1.5 mW by fast scanning (dwell time of 10μs), leading to phase transformation from amorphous to crystalline. After such laser treatment, the Si nanodisks became thermally stable, and then we started the nonlinear scattering measurements. The laser annealed Si can be poly-crystalline (poly-Si) or single crystalline (c-Si). Considering the similar optical properties between poly-Si and c-Si[1], we use the refractive index of crystalline silicon for all of our simulations.

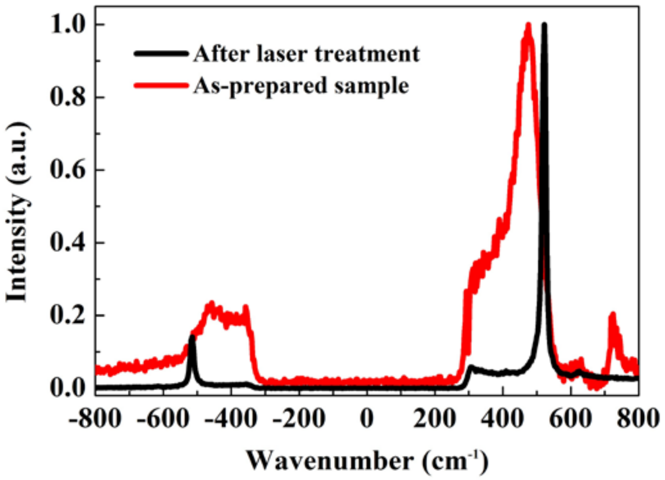

Supplementary Figure 1. Raman spectra of as-prepared Si nanodisks before and after the laser treatment.

Supplementary Note 2: Point spread function analysis of scattering images

Given the illumination beam with a certain intensity and Gaussian profile in space, the PSFs of a Si nanodisk in confocal reflectance images can be calculated based on nonlinear scattering response from a subwavelength object that is intensity dependent. The response function of the scattering intensity can be obtained from experiment results given in Fig. 1b in the main text and also in Supplementary Figure 2 (a). A series of theoretical simulations of scattering images with different excitation intensities are presented in the figures below, which nicely reproduces the experimental observations in Fig. 2d in the main text.

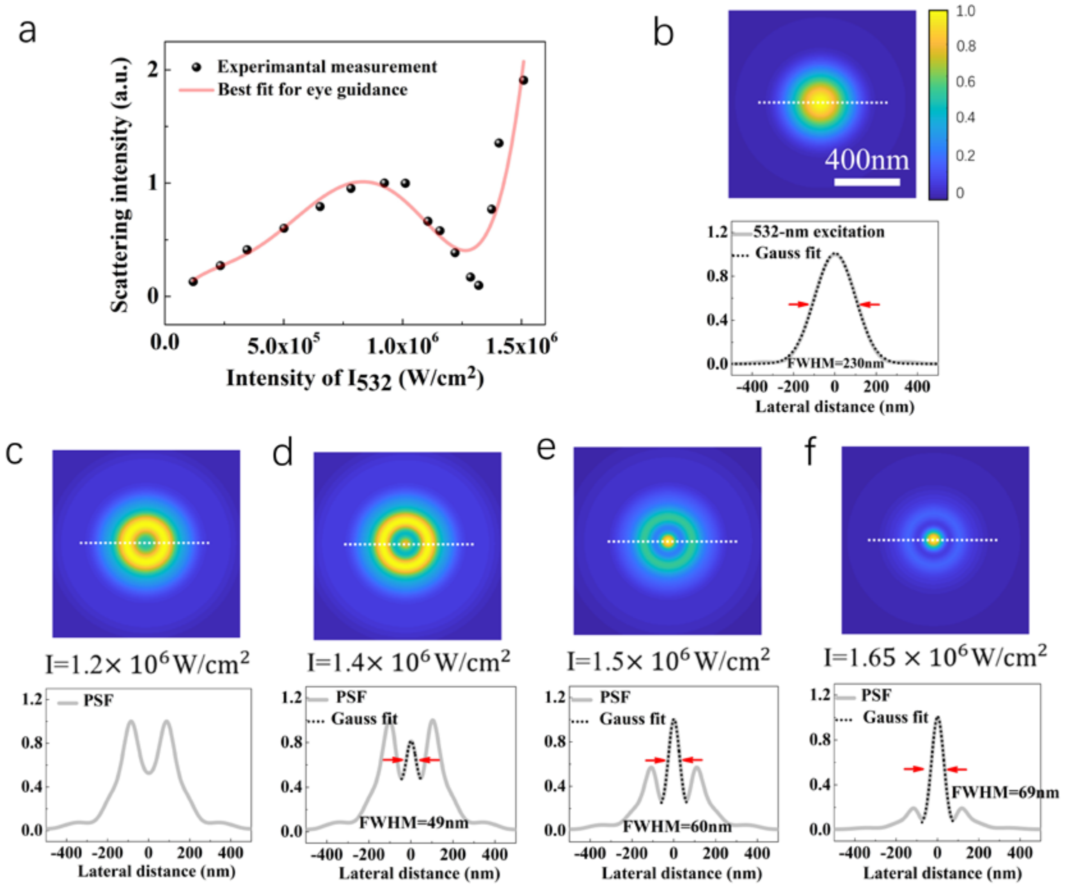

Supplementary Figure 2. Point spread function analysis of scattering images. (a) Measured nonlinear scattering dependence on the excitation intensity as well as the fit for eye guidance. (b) Gaussian distribution and the cross-section of the 532 nm excitation laser beam at the focal plane of an objective lens of NA=1.4. (c-f) Calculated nonlinear scattering images and corresponding cross-sections under different excitation intensities.

Supplementary Note 3: Reversibility of nonlinear scattering of Si nanodisks

Figure 2f in the main text confirms the reversibility of both scattering intensities and corresponding PSFs. Here the reversibility is further confirmed by examining the evolution of normalized scattering cross-sections with excitation intensities varied between low and high by several cycles.

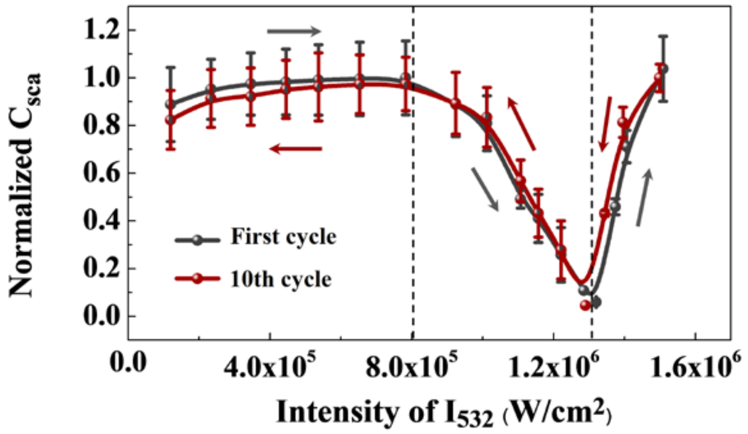

Supplementary Figure 3. Reversibility of normalized scattering cross-sections by varying the excitation intensities from low to high, and vice versa. The arrows in the figure indicate increasing (black) or decreasing (red) the excitation intensities. The error bars show the standard deviations of normalized scattering cross-sections according to statistics of twelve nanodisks.

Supplementary Note 4: Dark-field scattering of Si nanodisks

Dark-field scattering was measured using a reflectance dark-field microscope equipped with a spectrometer. The sample was illuminated by a halogen white light source using an objective lens (Olympus, MPlanFLN, 50×, NA=0.8). The reflected light was collected through the same lens and recorded by the spectrometer. Dark-field spectra before and after the nonlinear scattering are shown to manifest that the nonlinear scattering is fully reversible.

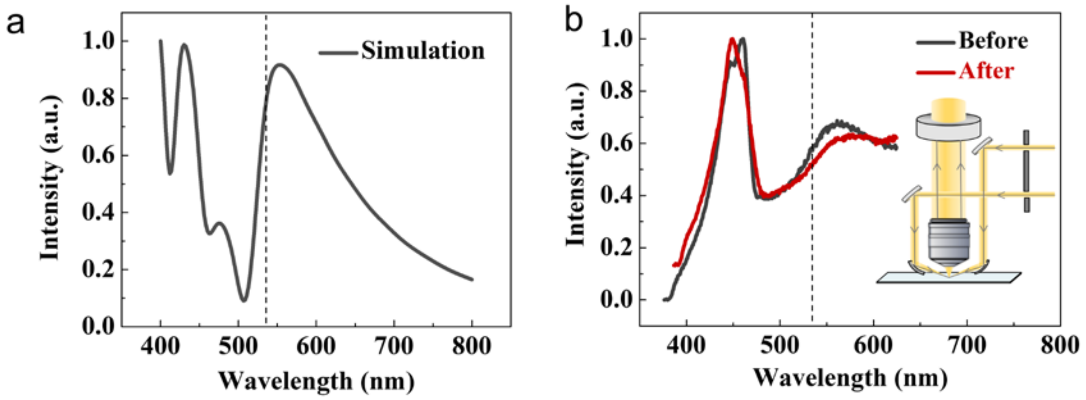

Supplementary Figure 4. Backward scattering spectrum of isolated Si nanodisks in FDTD simulation (a) and experimental dark-field spectra before and after the nonlinear scattering measurement (b). The dashed lines indicate the excitation wavelength position. The inset shows the schematic configuration of a reflectance dark-field microscope.

Supplementary Note 5: Raman thermometry for measuring temperature rises of Si nanodisks under optical heating

Raman scattering intensity is highly related to the vibrational density of states for a certain phonon energy $h\Omega$. Temperature-dependent phonon population of the vibrational states is given by the Bose-Einstein distribution function, with $\bar{n}$ for anti-Stokes and $\bar{n}+1$ for Stokes processes[2]:

$$\bar{n} = (\exp\frac{h\Omega}{k_{\mathrm{B}}T} - 1)^{-\mathbf{1}}$$

where h is the Plank constant, k_B is the Boltzmann constant and T is temperature. With this formula, we can derive the temperature by taking the ratio of anti-Stokes to Stokes intensity:

$$I_{\mathrm{A}}/I_{\mathrm{s}} = \bar{n}/(\bar{n}+1) = \exp\frac{-h\Omega}{k_{\mathrm{B}}T}$$

where $h = 6.626 \times 10^{-34}\mathrm{J \cdot s}$, $k_{\mathrm{B}} = 1.38 \times 10^{-23}\mathrm{J/K}$, and $\Omega = c\omega$ with $c = 3 \times 10^{10}\mathrm{cm/s}$, and ω is the Raman shift in $\mathrm{cm^{-1}}$. Anti-Stokes-to-Stokes ratio at the characteristic Raman shift ω =520 $\mathrm{cm^{-1}}$ was used to determine the temperature rise of Si with different light intensities.

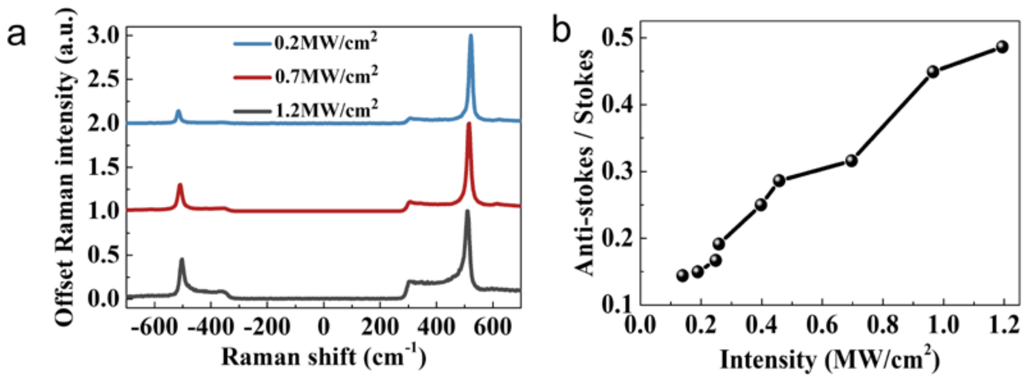

Supplementary Figure 5. (a) Raman spectra taken under three different irradiance intensities at the wavelength of 532 nm. The Stokes signal is normalized to 1 for better visualization of the ratio of anti-Stokes and Stokes lines. (b) Variation of anti-Stokes-to-Stokes ratio $I_{\mathrm{A}}/I_{\mathrm{s}}$ as a function of irradiance intensities.

Supplementary Note 6: Complex refractive index of silicon at elevated temperatures

Refractive indices of silicon in the visible wavelength range were taken from Jellison and Modine, which were measured using a two-channel spectroscopic polarization modulation ellipsometer[3]. Below the direct bandgap edge, the real part of the refractive index can be expressed as:

$$n(E,T) = n_0(E) + a(E)T,$$

with

$$n_0(E) = [4.565 + 97.3/(E_g^2 - E^2)]^{1/2},$$

$$a(E) = [-1.864 + 53.94/(E_g^2 - E^2)] \times 10^{-4}$$

where $E_g = 3.648eV$, E is the photon energy, and T is the temperature with unit of °C.

The imaginary part of the complex refractive index can be expressed as:

$$k(E,T) = k_0(E)\exp[T/T_0]$$

where $k_0(E) = -0.0805 + \exp[-3.1893 + 7.946/(E_g^2 - E^2)]$ and $T_0 = 369.9$°C.

With these empirical expressions, *n* and *k* are both parameterized as functions of temperature *T* and photon energy *E* for photon energies below the direct band edge of silicon. Although Jellison's work only gave measurements up to 490°C, the data extrapolation for higher temperatures was further corroborated with experimental data at 500°C, 700°C and 850 °C measured by Šik et al[4], validating its soundness. In addition, experimental measurements of temperature-dependent complex refractive index of single crystalline silicon with thickness of 170 nm was performed by ellipsometric methods. Restricted by the heater equipped in the ellipsometer, the measurement was only up to 400 °C. We present in Supplementary Figure 6 the temperature-dependent index reported in Refs. 3 and 4 together with our own measured data, showing reasonable agreement.

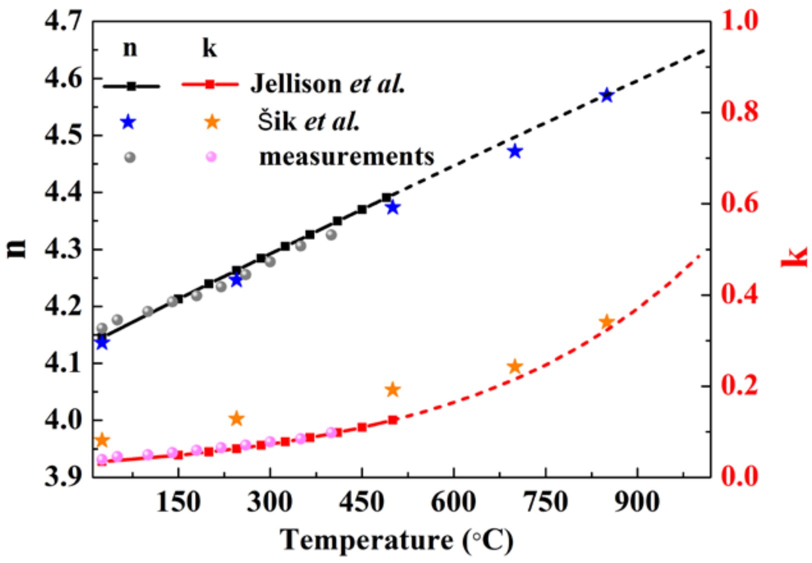

Supplementary Figure 6. Temperature-dependent refractive index at the wavelength of 532 nm from room temperature to 1000°C. Squares, stars and circles denote data given by Jellison et al[3], Šik et al[4] and our own measurements, respectively. The dash lines are the extrapolation based on the existing data.

Supplementary Note 7: Photothermal nonlinearity of unstructured bulk Si

Raman spectra of a standard Si wafer under the exposure of the 532 nm CW laser beam were measured. Following the same procedure for Si nanodisks, the temperature rises were calculated by measuring the ratio of anti-Stokes to Stokes intensity I_A/I_S in the Raman spectra.

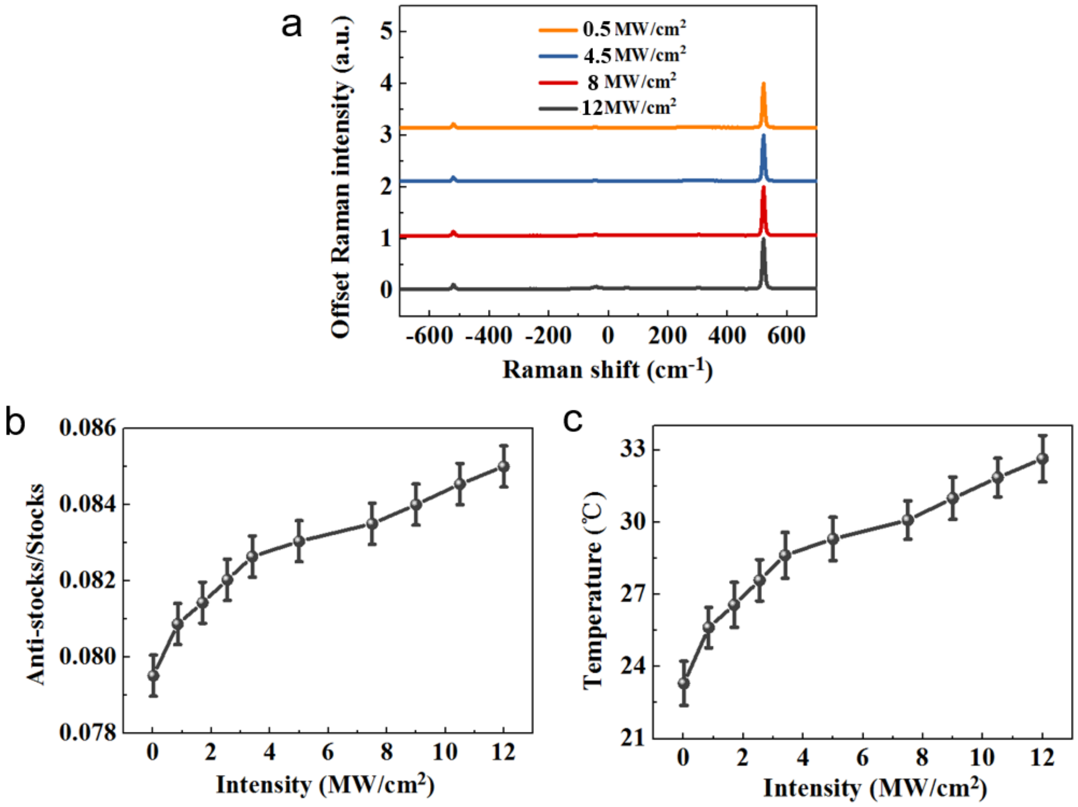

Supplementary Figure 7. Raman thermometry of unstructured bulk Si upon optical heating. (a) Raman spectra taken under four different laser intensities at the wavelength of 532 nm. The Stokes signal is normalized to be 1 for better visualization of the ratio of anti-Stokes and Stokes lines. (b) Variation of anti-Stokes-to-Stokes ratio I_A/I_S as a function of irradiance intensities. (c) Extracted temperature rises at different irradiance intensities. Error bars show the differences between the minimum and the maximum Raman intensity values.

It is clearly seen that bulk single crystalline Si has rather small change of I_A/I_S throughout the range of laser intensities, implying that there was less than 10℃ temperature rise. This corresponds to $\Delta n{\sim}0.005$ when laser intensity I is 12 MW/cm^2, leading to $n_{2,bulk@532nm} = \Delta n/I \approx 4 \times 10^{-4}$ cm^2/MW. Therefore, this result implies a three-order-of-magnitude enhanced photothermal nonlinearity by Si nanodisks supporting anapole states compared with the bulk Si.

Supplementary Note 8: Total, forward and backward scattering cross-sections at elevated temperatures

Previous study has reported the changes in the directional scattering of designed metasurfaces via thermal tuning[5]. In their study, temperature-dependent change of the refractive index of silicon can cause changes in scattering directionality, i.e. forward/backward scattering ratio. In order to clarify that in our case the modulation of the scattering cross-section originates from the photothermal tuning of anapole modes instead of redistribution of forward and backward radiations, we performed simulations accounting total scattering, forward scattering and backward scattering as we show below in Supplementary Figure 8. Herein for simulations of total, forward and backward scattering, we use two half-open box monitors which collect all the scattering fields of forward and backward 2π solid angles. This yields the sum of C_{scaF} and C_{scaB} equals to total C_{sca}. As can be seen in Supplementary Figure 8, in all the cases, the excitation at the wavelength of 532 nm acts as photothermal tuning close to the anapole mode, and the scattering cross-sections all experience suppression at the beginning and recovering with temperature rises in similar trends. It should be noted that in the calculations of backward scattering C_{scaB} in Figs. 3e-g in the main text, a plane monitor was employed. The plane monitor was positioned at 400 nm from the simulation center, with a monitor size of 1900 nm by 1900 nm to collect most of the backward scattering power. Such settings are based on the consideration of matching the maximum collection angle of the objective lens (NA = 1.4) used in our experiments.

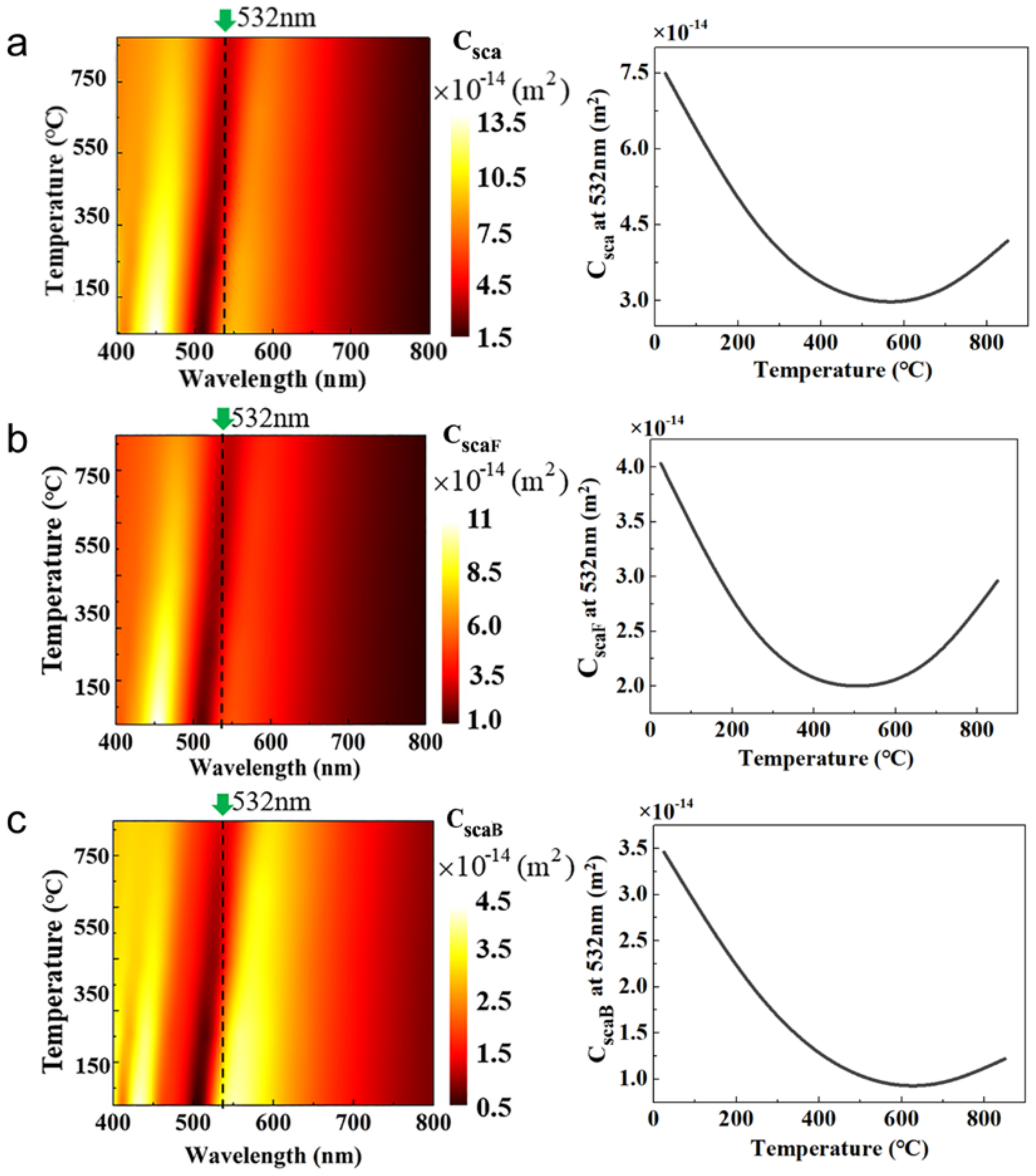

Supplementary Figure 8. Simulated scattering cross-sections in different configurations. Simulation maps for total scattering (a) and forward scattering (b) and backscattering (c). The right panels plot the variations of scattering cross-sections at the wavelength of 532 nm at different temperatures.

Supplementary Note 9: Photothermal induced nonlinear scattering of Si nanodisks with different sizes

Photothermal nonlinearity by two other sized Si nanodisks was calculated to verify the important role played by anapole modes. Photothermal tuning of backward scattering spectra of Si nanodisks of diameter of 170 nm and 230 nm are shown in Supplementary Figure 9-A. For a smaller-sized nanodisk (D=170 nm), it is seen that the overall photothermal tuning occurs near its ED mode. We show that ED-mediated process presents much weaker photothermal nonlinearity by the fact of temperature increasing less than 200℃ (Supplementary Figure 9-B (d)). The induced refractive index change is up to $\Delta n \sim 0.1$ for photothermal tuning close to the ED mode. This is five times smaller than photothermal nonlinearity driven by the anapole mode. Meanwhile, the backward scattering cross-section keeps almost unchanged (slightly decreases from about 1.65 to 1.62×10^{-14}m^2, less than 2%, shown in the shadow range in Figure 9-B (e)) within elevated temperatures. Therefore, even employing a laser intensity up to 1.5 MW/cm^2, the Si nanodisks present negligible nonlinearity (Figure 9-B (f)). On the contrary, for larger-diameter nanodisk (D=230 nm), elevated temperatures induce the anapole red-shifting away from the exciting wavelength, leading to a monotonical increase of backward scattering cross sections. Thus, a sharp RSS is achieved (Figure 9-B (g-i)).

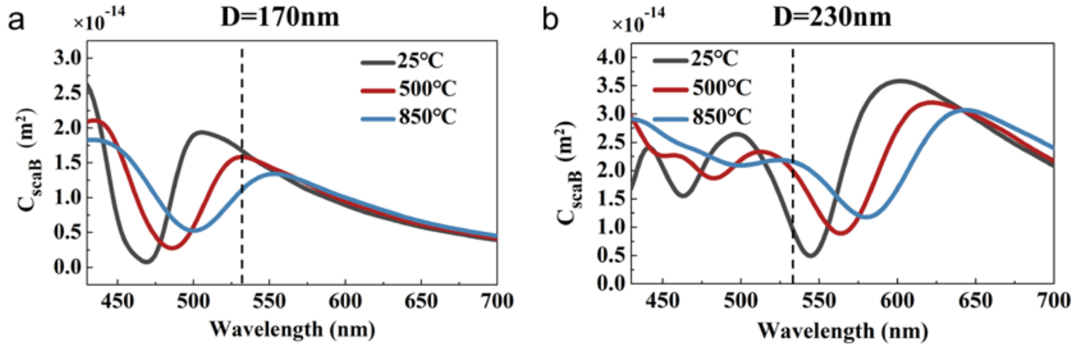

Supplementary Figure 9-A. Photothermal tuning of backward scattering spectra of Si nanodisks of different sizes.

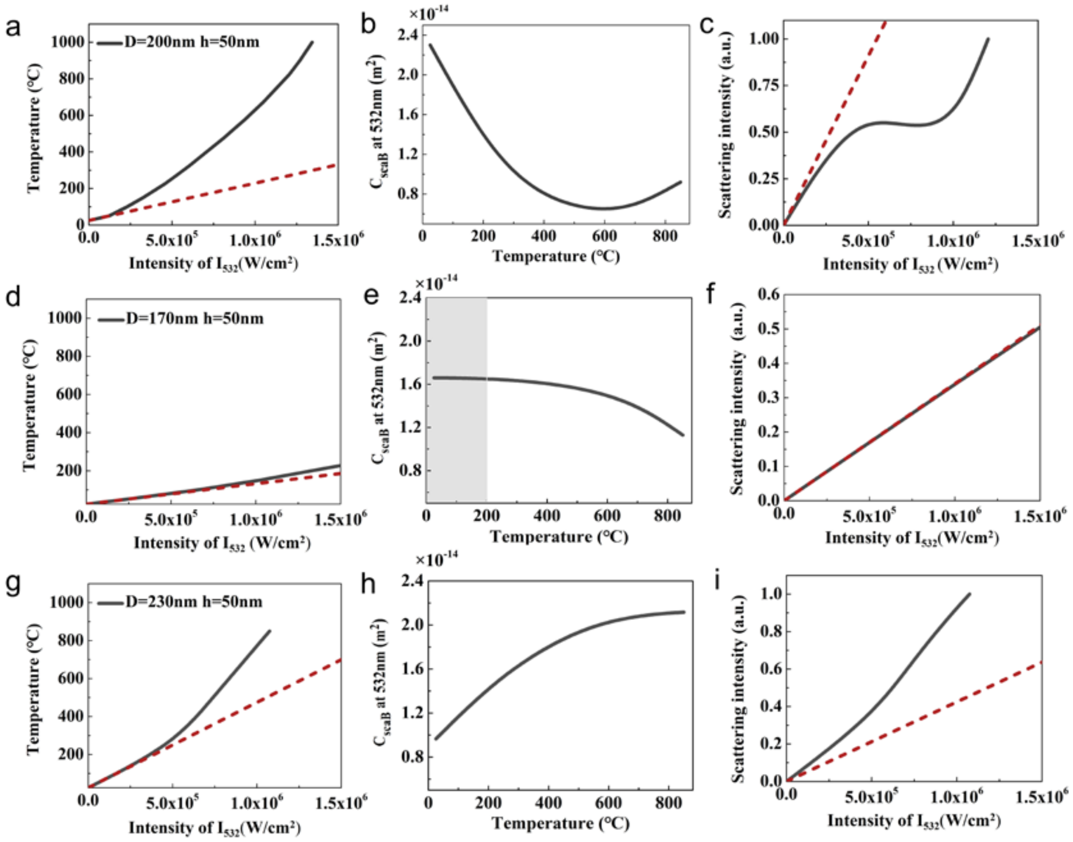

Supplementary Figure 9-B. Simulation of temperature variations and corresponding nonlinear scattering response of Si nanodisks with different sizes. The top panels (a)-(c) show the results of nanodisks with D=200 nm and h=50 nm, same as Fig. 3(c), 3(g), and 3(h) in the main text. The middle panels (d)-(f) show for nanodisks with D=170 nm and h=50 nm, whilst the bottom panels (g)-(i) show for nanodisks with D=230 nm and h=50 nm. (a), (d) and (g) are the temperature rises under the given excitation intensity. (b), (e) and (h) are the scattering cross-section modulations when increasing the temperature. Note that photothermal tuning near the ED mode results in very small changes in backward scattering cross-sections (marked by the shadow range in (e)). By combining the above two sets of figures, (c), (f) and (i) present the resulting simulated nonlinear scattering behaviors. Dash lines denote the linear trends without taking the photothermal nonlinearity into account.

Supplementary Note 10: Characterizations and more experimental results of nonlinear scattering for periodic Si nanodisk arrays

The morphology of the dense arrays of Si nanodisks was characterized by AFM. The AFM image in Supplementary Figure 10 (a) shows the large-scale uniformity of the prepared Si nanodisks. Figure 10 (b) presents that the heights of the Si nanodisks are 50 nm±2 nm. The holes shown in the AFM image (zoom-in region in Figure 10 (c)) are defects caused by the vacancy of self-assembled polystyrene spheres during the colloidal lithography, where the glass substrate is exposed to the etching directly where etching rate is faster at positions without sphere masks. Therefore, the defect region is etched about 60 nm in depth in the glass substrate.

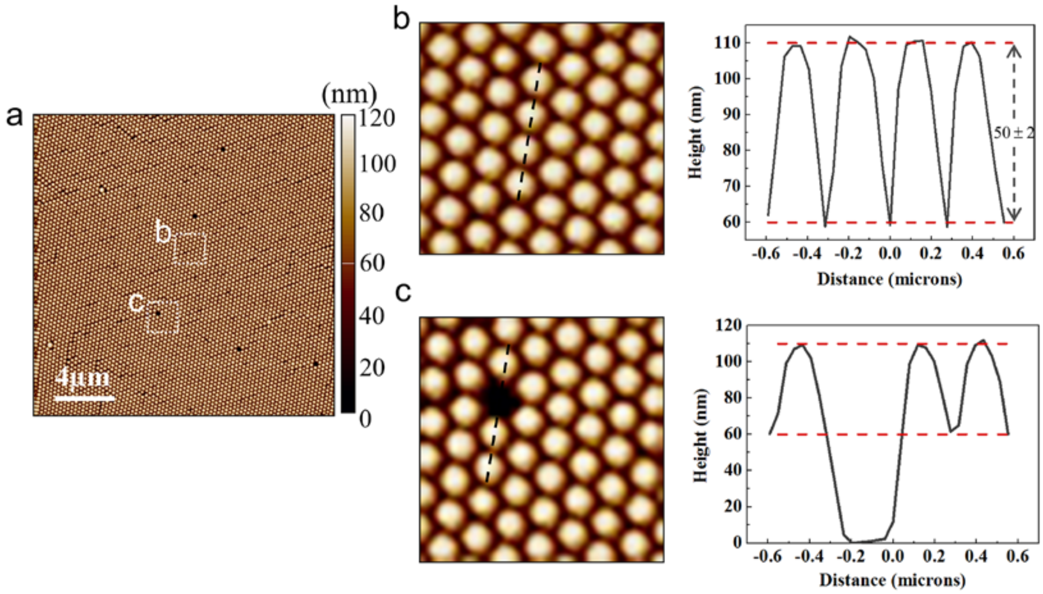

Supplementary Figure 10. AFM Characterization of the Si nanodisk array sample. (a) AFM image and zoom-in images (b and c) of Si nanodisk arrays with D=200 nm and h=50 nm, Scale bar: 4μm. Zoom-in images for a region containing uniformly distributed Si nanodisks (b) and a region containing defects (c). The right panels are cross-sections to show the height of the nanodisks.

Supplementary Note 11: Confocal reflectance images of Si nanodisks in dense arrays and correlations with SEM images

The confocal reflectance images of Si nanodisks in dense arrays under different irradiance intensities are shown in Supplementary Figure 11, which clearly manifest the evolution of PSFs from SS to RSS. The optical localization image of such Si nanodisk arrays through photothermal nonlinearity is highly reproducible, which was confirmed by the SEM images before and after the nonlinear scattering measurement (Supplementary Figure 12).

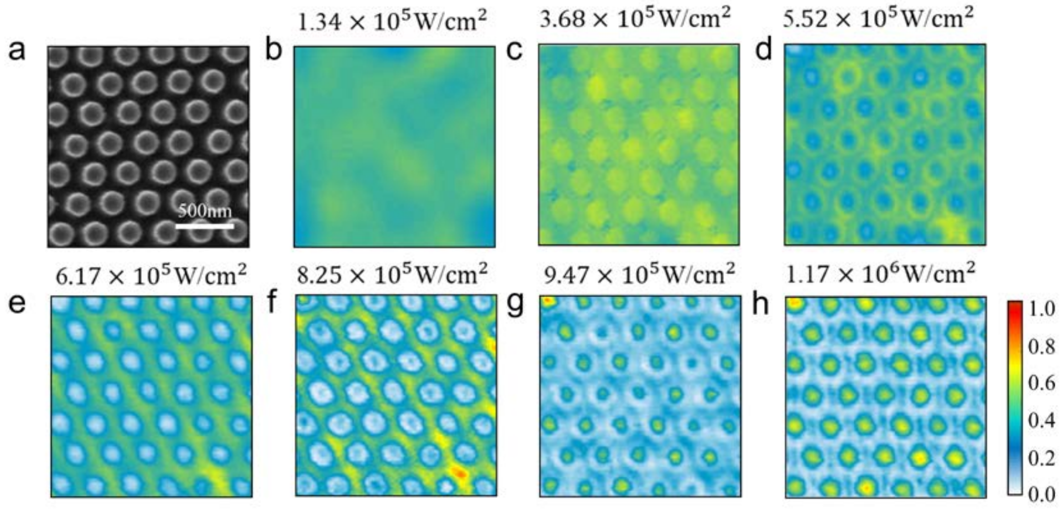

Supplementary Figure 11. Confocal reflectance images of Si nanodisk arrays at different stages of nonlinear scattering. (a) to (h) The progressive evolution of PSFs with increasing excitation intensities. The sample area is the same with what we show in the main text in Fig.4b.

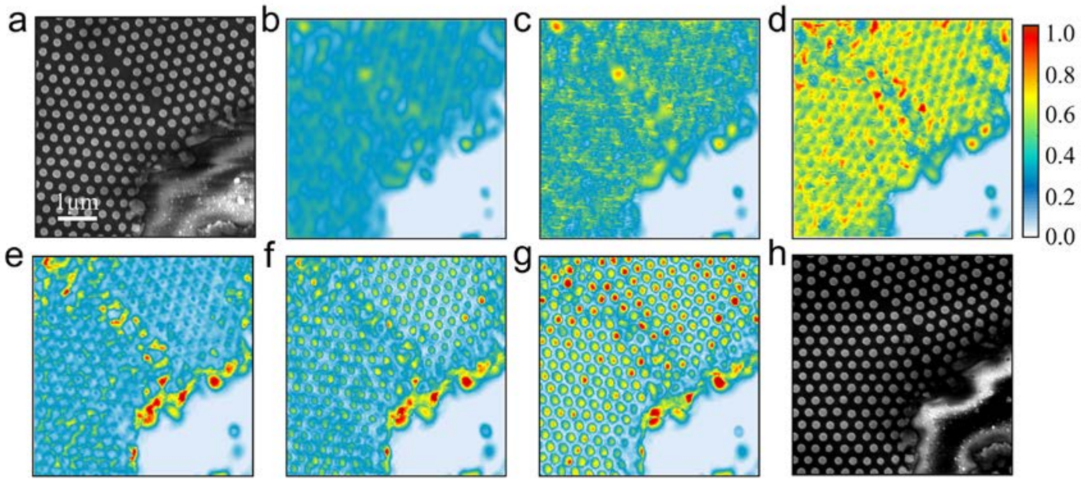

Supplementary Figure S12. Collections of confocal reflectance images of Si nanodisk arrays at different stages of nonlinear scattering. Correlations between SEM images and optical images are unambiguously demonstrated by imaging a sample area containing defects. (a) SEM image of the sample area taken before imaging by photothermal nonlinear scattering. (b)-(h) show confocal reflectance images of Si nanodisk arrays acquired at different irradiance intensities. (h) is the SEM image we taken after the optical measurement, which verifies that the Si nanodisks are well preserved without any photo-damage or deformation.

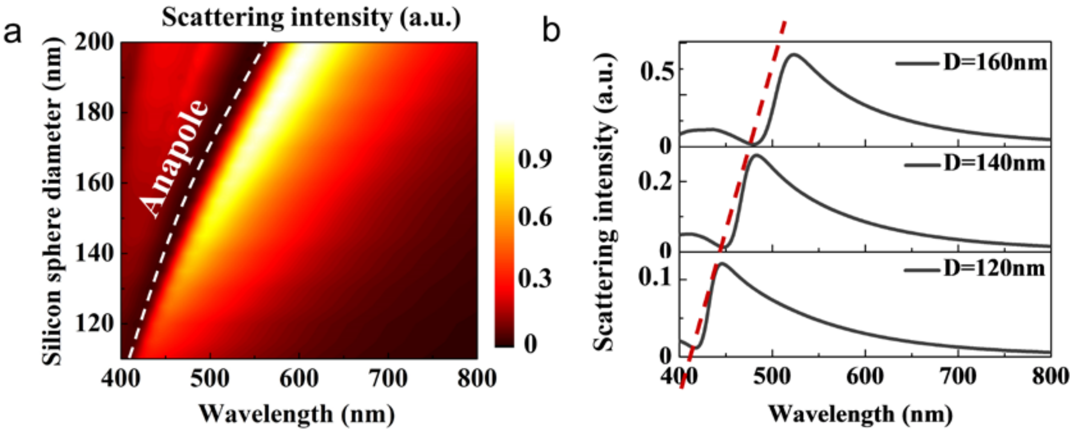

Supplementary Figure 13. Simulation map of total scattering spectra of Si nanospheres with various sizes. Dash lines indicate the scattering valleys (anapole state) varying with diameters of nanospheres.

Supplementary References

1. Mirshafieyan, S. S. & Guo, J. Silicon colors: spectral selective perfect light absorption in single layer silicon films on aluminum surface and its thermal tunability. *Opt Express* **22**, 31545-31554 (2014).
2. Bhusari, D. M., Kumbhar, A. S. & Kshirsagar, S. T. Temperature-dependent Raman studies of hydrogenated-amorphous-silicon films. *Phys Rev B* **47**, 6460-6464 (1993).
3. Jellison, G. E. & Modine, F. A. Optical functions of silicon at elevated temperatures. *Journal of Applied Physics* **76**, 3758-3761 (1994).
4. Šik, J., Hora, J. & Humlíček, J. Optical functions of silicon at high temperatures. *Journal of Applied Physics* **84**, 6291-6298 (1998).
5. Rahmani, M. et al. Reversible Thermal Tuning of All-Dielectric Metasurfaces. *Adv Funct Mater* **27**, 1700580 (2017).
