# Supplementary material for: Anapole mediated giant photothermal nonlinearity in nanostructured silicon
Source: Nat Commun. 2020 Jun 15;11:3027. doi: 10.1038/s41467-020-16845-x (PMC7296001; doi:10.1038/s41467-020-16845-x)
Supplement: Supplementary file 3 — Description of Additional Supplementary Files [file 41467_2020_16845_MOESM3_ESM.pdf]

Description of Additional Supplementary Files

File Name: Supplementary Movie 1

Description: A typical example of the PSF evolution by increasing the irradiance intensity. The sample is densely packed Si nanodisk arrays which was raster scanned by a CW laser beam at the wavelength of 532 nm.
